# Maternal adverse childhood experiences impact fetal adrenal volume in a sex-specific manner

**DOI:** 10.1186/s13293-023-00492-0

**Published:** 2023-02-17

**Authors:** Korrina A. Duffy, Mary D. Sammel, Rachel L. Johnson, Deborah R. Kim, Eileen Y. Wang, Grace Ewing, Liisa Hantsoo, Sara L. Kornfield, Tracy L. Bale, C. Neill Epperson

**Affiliations:** 1 Department of Psychiatry, University of Colorado School of Medicine Anschutz Medical Campus, Aurora, CO USA; 2 Department of Psychiatry, University of Colorado – Anschutz Medical Campus, 1890 N. Revere Court, Aurora, CO 80045 USA; 3 Department of Biostatistics and Informatics, University of Colorado School of Public Health – Anschutz Medical Campus, Aurora, CO USA; 4 Department of Psychiatry, University of Pennsylvania Perelman School of Medicine, Philadelphia, PA USA; 5 Department of Obstetrics and Gynecology, University of Pennsylvania Perelman School of Medicine, Philadelphia, PA USA; 6 University of New England College of Osteopathic Medicine, Biddeford, ME USA; 7 Department of Psychiatry and Behavioral Sciences, The Johns Hopkins University School of Medicine, Baltimore, MD USA; 8 Department of Family Medicine, University of Colorado School of Medicine – Anschutz Medical Campus, Aurora, CO USA

**Keywords:** Maternal early life stress, Preconception stress, Fetal hypothalamic–pituitary–adrenal axis, Fetal adrenal gland, Dysmasculinization, Sex differences

## Abstract

**Background:**

The mechanisms by which parental early life stress can be transmitted to the next generation, in some cases in a sex-specific manner, are unclear. Maternal preconception stress may increase susceptibility to suboptimal health outcomes via in utero programming of the fetal hypothalamic–pituitary–adrenal (HPA) axis.

**Methods:**

We recruited healthy pregnant women (*N* = 147), dichotomized into low (0 or 1) and high (2+) adverse childhood experience (ACE) groups based on the ACE Questionnaire, to test the hypothesis that maternal ACE history influences fetal adrenal development in a sex-specific manner. At a mean (standard deviation) of 21.5 (1.4) and 29.5 (1.4) weeks gestation, participants underwent three-dimensional ultrasounds to measure fetal adrenal volume, adjusting for fetal body weight (_wa_FAV).

**Results:**

At ultrasound 1, _wa_FAV was smaller in high versus low ACE males (*b* = − 0.17; *z* = − 3.75; *p* < .001), but females did not differ significantly by maternal ACE group (*b* = 0.09; z = 1.72; *p* = .086). Compared to low ACE males, _wa_FAV was smaller for low (*b* = − 0.20; *z* = − 4.10; *p* < .001) and high ACE females (*b* = − 0.11; *z* = 2.16; *p* = .031); however, high ACE males did not differ from low (*b* = 0.03; *z* = .57; *p* = .570) or high ACE females (*b* = − 0.06; *z* = − 1.29; *p* = .196). At ultrasound 2, _wa_FAV did not differ significantly between any maternal ACE/offspring sex subgroups (*p*s ≥ .055). Perceived stress did not differ between maternal ACE groups at baseline, ultrasound 1, or ultrasound 2 (*p*s ≥ .148).

**Conclusions:**

We observed a significant impact of high maternal ACE history on _wa_FAV, a proxy for fetal adrenal development, but only in males. Our observation that the _wa_FAV in males of mothers with a high ACE history did not differ from the _wa_FAV of females extends preclinical research demonstrating a dysmasculinizing effect of gestational stress on a range of offspring outcomes. Future studies investigating intergenerational transmission of stress should consider the influence of maternal preconception stress on offspring outcomes.

**Supplementary Information:**

The online version contains supplementary material available at 10.1186/s13293-023-00492-0.

## Highlights

- Three-dimensional ultrasound serves as a non-invasive way to measure fetal adrenal volume as a proxy for development of the fetal hypothalamic–pituitary–adrenal axis.
- Weight-adjusted fetal adrenal volume (_wa_FAV) differed between subgroups based on offspring sex and the mother’s history of adverse childhood experiences (ACEs).
- In males of mothers with a high ACE history, _wa_FAV was significantly smaller than in males of mothers with a low ACE history but indistinguishable from the _wa_FAV of females from either maternal ACE group; however, males of mothers with a low ACE history had larger _wa_FAV than females from either maternal ACE group.
- These findings suggest male vulnerability to dysmasculinization of _wa_FAV in response to maternal preconception stress in line with previous animal studies showing a dysmasculinizing effect of gestational stress on a range of offspring outcomes.

**Supplementary Information:**

The online version contains supplementary material available at 10.1186/s13293-023-00492-0.

## Introduction

Mothers with a history of adverse childhood experiences (ACEs)—such as abuse, neglect, and family dysfunction—are more likely to have offspring with poorer mental health outcomes across the lifespan [1, 2]. Although rodent models suggest that maternal preconception stress impacts offspring through epigenetic changes to oocytes or in utero exposures [3–6], no studies have tested the effect of maternal ACEs on human fetal development despite its impact being theoretically plausible [7]. Presumably, dysregulated maternal stress physiology associated with maternal ACEs [8–15] could alter communication with the maternal–placental–fetal unit in a manner that leads to intergenerational transmission of a stress phenotype [16–20].

Although no human or animal studies have tested for an effect of maternal preconception stress (occurring before pregnancy) on fetal hypothalamic–pituitary–adrenal (HPA) axis, studies have examined this in the context of prenatal stress (occurring during pregnancy) or hormonal proxies of prenatal stress (glucocorticoid administration). In humans, ACEs impact maternal glucocorticoids in complex ways during pregnancy [17] and increase placental corticotropin releasing hormone (pCRH) [16]. In turn, higher maternal cortisol and placental pCRH levels during gestation have been linked to fetal growth restriction [21], earlier birth [22], lower birthweight [23], and enhanced offspring stress response [24, 25]. Although maternal cortisol is largely inactivated by placental 11β-hydroxysteroid dehydrogenase type 2 (11β-HSD-2), maternal cortisol levels still explain a third of the variation in fetal cortisol levels [26]. In animal models, maternal HPA axis function altered by administration of either ACTH or dexamethasone provide direct evidence of the impact of high glucocorticoid exposure on fetal brain regions involved in stress responsivity [18, 19] and the fetal HPA axis, including the weight of the adrenal glands [18]. In an animal model of prenatal stress focusing exclusively on male offspring, male rats of stress exposed mothers exhibited decreased weight of the fetal adrenal glands relative to control males [20].

To our knowledge, the current study is the first to examine the volume of the human fetal adrenal gland as a proxy for the impact of maternal ACEs on fetal HPA axis development. We chose to focus on the fetal adrenal for three reasons. First, the adrenals are an essential stress-responsive organ of the HPA axis and the earliest and fastest growing organ in the fetal HPA axis [27]. Second, in animal models, its volume varies based on gestational stress exposure, at least in males [20], suggesting that high maternal ACEs and their potential to disrupt maternal HPA axis function during gestation could impact fetal adrenal development. Third, technological advances in three-dimensional (3-D) ultrasound have made it possible to measure fetal adrenal volume (FAV) noninvasively in humans [28, 29], with good intra- and interrater reliability [30].

We designed this study (1) to evaluate the relationship between maternal ACEs and fetal body weight-adjusted FAV (_wa_FAV) and (2) to consider fetal sex as a potential moderator. In animal models, the impact of gestational stress exposure on fetal adrenal gland development has not been studied in females and, in humans, prenatal stress exposure impacts offspring outcomes in a sex-dependent manner (e.g., [23, 31–38, 40, 42]). Our sample included psychiatrically and medically healthy women, with the intention of isolating the effect of maternal ACEs on _wa_FAV without the confounding impact of maternal mental health or medical problems during gestation. Despite the absence of robust data on the relationship between maternal childhood adversity and fetal outcomes, we predicted that the fetuses of pregnant women with higher exposure to childhood adversity versus those low or no exposure would exhibit differences in _wa_FAV—particularly for males, given a review of clinical studies suggesting greater male vulnerability to gestational stress [41].

## Methods and materials

### Participant recruitment and screening

Women 18+ years old (at 8–17 weeks gestation) were recruited and screened while waiting for a perinatal care visit at three obstetrics and gynecology clinics affiliated with an academic health system. Participants were required to have a singleton pregnancy, be fluent in written and spoken English, and be willing to give written informed consent. Exclusions included reporting a serious medical or neurological illness, an active psychiatric illness, or a history of drug or alcohol abuse within the previous two years; feeling sad most days over the past two weeks; using steroid drugs or antihypertensives during pregnancy; having a history of fetal loss or preterm birth; or having a known abnormality with the current pregnancy or fetus. Eligible women were invited to undergo full screening for a longitudinal study that spanned pregnancy to 6 months postpartum. We recruited equal numbers of women reporting 0–1 and 2 + ACEs based on previous research demonstrating that 2+ ACEs increase risk for preterm birth [42], depression [43], cognitive complaints [44], and gut microbiota associated with inflammation [45] in women during reproductive transitions. All analyses in this paper focus on the sample of 147 participants who had _wa_FAV data from at least one of the two ultrasounds (both ultrasounds: *N* = 128; only one ultrasound: *N* = 19; first ultrasound: *N* = 140; second ultrasound: *N* = 138), which occurred on average (SD) at 21.5 (1.4) weeks gestation and 29.5 (1.4) weeks gestation. The timepoints were chosen based on precedent for measuring fetal adrenal gland size [29, 46], physiological relevance as indicated by previous studies [47, 48], and feasibility constrained by when women typically present for their first perinatal visit as well as when FAV can be most accurately visualized by 3D ultrasound. The University of Pennsylvania’s institutional review board approved all research activities. Our data are publicly available at Open Science Framework (https://osf.io/bp7as/). For participant characteristics, see Table 1.

**Table 1:** Participant characteristics overall and by maternal ACE group

	*N* with data	Overall (*N* = 147)	Low ACE (*N* = 71)	High ACE (*N* = 76)	*P*-value
Maternal demographics					
Maternal age	147	28.4 (5.3)	28.7 (5.0)	28.1 (5.5)	.480
BMI	143				**.012**
Normal/underweight		72 (49%)	43 (60.6%)	29 (38.2%)	
Overweight/obese		71 (48.3%)	27 (38%)	44 (57.9%)	
Missing		4 (2.7%)	1 (1.4%)	3 (3.9%)	
Race	147				**.032**
African American/Black		72 (49.0%)	28 (39.4%)	44 (57.9%)	
Caucasian/other		75 (51.0%)	43 (60.6%)	32 (42.1%)	
Ethnicity	147				1
Non-Hispanic/unknown		136 (92.5%)	66 (93.0%)	70 (92.1%)	
Hispanic		11 (7.5%)	5 (7.0%)	6 (7.9%)	
Marital status	147				**.008**
Married/domestic partner		76 (51.7%)	45 (63.4%)	31 (40.8%)	
Single/divorced		71 (48.3%)	26 (36.6%)	45 (59.2%)	
Education	147				**.001**
High school education or less		37 (25.2%)	14 (19.7%)	23 (30.3%)	
Some education after high school		40 (27.2%)	12 (16.9%)	28 (36.8%)	
College degree or more		70 (47.6%)	45 (63.4%)	25 (32.9%)	
Income	145				** < .001**
$25K or less		52 (35.4%)	20 (28.2%)	32 (42.1%)	
$25K to $75K		38 (25.9%)	11 (15.5%)	27 (35.5%)	
$75K or more		55 (37.4%)	38 (53.5%)	17 (22.4%)	
Parity	120	0.7 (0.9)	0.6 (0.7)	0.8 (1.0)	.175
Maternal psychological measures					
Baseline PSS score	145	14.0 (6.3)	13.7 (5.7)	14.3 (6.8)	.562
Ultrasound 1 PSS score	144	10.8 (6.7)	10.6 (6.0)	11.6 (7.1)	.148
Ultrasound 2 PSS score	143	10.0 (6.8)	9.7 (6.8)	10.4 (6.9)	.504
Baseline EPDS score	146	4.4 (4.1)	3.7 (3.3)	5.2 (4.6)	**.023**
Baseline STAI state score	147	28.9 (9.4)	30.2 (10.3)	27.5 (8.2)	.080
Baseline STAI trait score	147	31.6 (8.4)	33.2 (9.7)	29.9 (6.4)	**.016**
Offspring demographics					
Offspring sex	147				.070
Male		78 (53.1%)	32 (45.1%)	46 (60.5%)	
Female		69 (46.9%)	39 (54.9%)	30 (39.5%)	
Gestational age (weeks)					
Ultrasound 1	142	21.5 (1.4)	21.8 (1.4)	21.3 (1.4)	**.039**
Ultrasound 2	141	29.5 (1.4)	29.8 (1.3)	29.2 (1.4)	**.018**
Birthweight (g)	115	3270 (515)	3220 (557)	3317 (471)	.317

Maternal and offspring characteristics for women who had at least one ultrasound measuring fetal adrenal volume. Continuous variables are summarized with means and standard deviations, and differences between maternal ACE groups are tested using two-sample *t*-tests. Categorical variables are summarized with frequencies and percentages, and differences between maternal ACE groups are tested using Fisher’s exact tests. Tests with significant *p*-values are bolded. Values are summarized overall and stratified by maternal adverse childhood experiences (ACE) group: low = 0–1 ACEs; high = 2 + ACEs. PSS = Perceived Stress Scale; EPDS = Edinburgh Postnatal Depression Scale; STAI = State-Trait Anxiety Inventory

### Study procedures

#### ***Self-report measures at initial and full screening (Table ******2******)***

**Table 2:** Descriptions of psychological measures

Adverse Childhood Experiences Questionnaire (ACE-Q)	Participants completed the 10-item *Adverse Childhood Experiences Questionnaire* (ACE-Q) [49], which assessed exposures that occurred before age 18: abuse (emotional, physical, and sexual), neglect (emotional and physical), and household dysfunction (parental separation or divorce, household domestic violence, household substance abuse, parental mental illness, and member of household imprisoned). Each exposure counted as one point. ACE scores were computed by summing all exposures (0–10)
Perceived Stress Scale (PSS)	Participants completed the *Perceived Stress Scale* (PSS) [54], which assessed how unpredictable, uncontrollable, overloaded, and stressful they perceived their life to be. On 10 questions related to perceived stress, participants indicated how often they felt or thought a certain way over the last month (0 = *never*, 1 = *almost never*, 2 = *sometimes*, 3 = *fairly often*, 4 = *very often*). PSS scores were calculated by summing all items
Edinburgh Postnatal Depression Scale (EPDS)	Participants completed the *Edinburgh Postnatal Depression Scale* (EPDS) [50], which assessed depressive symptomatology over the past week. Although the scale was originally developed to measure depressive symptoms in postpartum women, it has been validated for use in antepartum women as well [83, 84]. On 10 items measuring depressive symptoms, participants indicated how often they felt or thought a certain way (e.g., “I have been sad or miserable”) on a four-point scale, with higher scores indicating greater frequency of depressive symptoms. EPDS scores were calculated by summing all items
Spielberger State-Trait Anxiety Inventory (STAI)	Participants completed the *Spielberger State‐Trait Anxiety Inventory* (STAI) [53]. The trait anxiety subscale (STAI-T) asked participants to report how they generally felt on 20 items related to their general anxiety, e.g., “some unimportant thought runs through my mind and bothers me” (1 = *almost never*, 2 = *sometimes*, 3 = *often*, 4 = *almost always*). The state anxiety subscale (STAI-S) asked participants to report how they felt at the moment on 20 items related to their current anxiety, e.g., “I feel nervous” (1 = *not at all*, 2 = *somewhat*, 3 = *moderately so*, 4 = *very much so*). Trait and state anxiety scores were calculated by summing all items

At initial screening, participants completed the *Adverse Childhood Experiences Questionnaire* (ACE-Q) [49], the *Edinburgh Postnatal Depression Scale* (EPDS) [50], and a questionnaire assessing demographics as well as medical/obstetrics history. At full screening, participants were administered the *Structured Clinical Interview* for *DSM-IV-TR* (SCID) [51] to rule out current psychiatric illness and the *Hamilton Depression Rating Scale* (HAM-D) [52] to assess subclinical depressive symptoms. At this visit, they also completed the EPDS and the *Spielberger State‐Trait Anxiety Inventory* (STAI) [53]. At the full screening and at each ultrasound visit, participants completed the *Perceived Stress Scale* (PSS) [54].

#### Fetal adrenal volume

For each ultrasound, either the right or left adrenal gland was selected to be measured based upon which adrenal had the clearest boundaries. Two raters (GE, EW) then measured the adrenal gland in replicate, repeating the measurement either two or three times. The replicates were averaged within rater and then between raters. The intraclass correlation coefficients were excellent for both ultrasounds within rater (ultrasound 1, rater 1: 0.899, 95% CI: [0.875, 0.920]; ultrasound 1, rater 2: 0.902, 95% CI: [0.878, 0.923]; ultrasound 2, rater 1: 0.859, 95% CI: [0.825, 0.888]; ultrasound 2, rater 2: 0.912 95% CI: [0.891, 0.931]) and between raters (ultrasound 1: 0.973, 95% CI: [0.964, 0.980]; ultrasound 2: 0.979, 95% CI: [0.971, 0.984]). Fetal body weight was estimated using a formula that included abdominal circumference, femur length, and head circumference [55]. We then divided FAV (cm^3^) by fetal body weight (kg) to yield weight-adjusted FAV (_wa_FAV cm^3^/kg). See Additional file 1: Tables S1 and S2 for gestational age, adrenal volume, fetal body weight, and body weight-adjusted fetal adrenal volume data stratified by maternal ACE group and offspring sex for ultrasounds 1 and 2. See Additional file 1: Figures S1 and S2 in supplementary materials for histograms of fetal adrenal volumes overall and separately by sex. For details on 3-D ultrasound methods, see supplementary materials and Kim et al. [30]. During a postpartum visit, fetal sex was confirmed.

### Statistical plan

#### Rationale for ACE as a dichotomous variable

Through visualization of the data, we determined that the relationship between maternal ACE and _wa_FAV was not linear. To assess the validity of our a priori hypothesis that 2+ ACEs would indicate the risk group in this sample, we tested whether continuous ACEs versus dichotomized ACEs (at all possible cutoff points) produced the best fitting model. As such, we modeled the three-way interaction of maternal ACEs, offspring sex (male vs. female), and time (ultrasound 1 vs. 2) on _wa_FAV in a linear mixed effects model with a random intercept for each participant. We found that dichotomizing ACE by a low (0–1 ACEs) and high (2+ ACEs) group led to the best fitting model based on having the lowest Akaike Information Criterion (AIC) (Additional file 1: Table S3).

#### Testing for covariates

We included the maternal and offspring demographic variables (listed in Table 1) as covariates in subsequent models if the covariate was associated with maternal ACE group and at least one of the ultrasound measures of _wa_FAV with a *p* < 0.10. Although offspring sex is listed under offspring demographics in Table 1, we did not consider it for inclusion as a covariate given that we included it as a moderator.

#### Modeling the effect of maternal ACE group, offspring sex, and time on fetal adrenal volume

To determine whether a three-way interaction existed between maternal ACE group (low vs. high), offspring sex (male vs. female), and time (ultrasound 1 vs. 2) on _wa_FAV, we modeled the effect of maternal ACE group, offspring sex, time, and all combinations of their interactions using a linear mixed effects model with a random intercept for each participant. This allowed us to model data even if participants were missing one of the two ultrasounds. Within this larger model, we tested for pairwise comparisons of the offspring sex/maternal ACE subgroups at ultrasounds 1 and 2 as these were our primary outcomes of interest.

## Results

### Sample characteristics

The sample consisted of 147 participants (age: *M* = 28.4, SD = 5.3), roughly balanced between the low and high maternal ACE groups (low ACE: *N* = 71; high ACE: *N* = 76), who differed on several participant characteristics, such as BMI, race, marital status, education, and income (see Table 1). Compared to the low ACE group, the high ACE group had lower trait anxiety (STAI-trait; *t* = 2.44; df = 130.97; *p* = 0.016) but higher depressive symptoms (EPDS; *t* = 2.30; df = 135.76; *p* = 0.023)—although their average was far below the cutoff of ≥ 14 that indicates possible clinical depression [56]. Perceived stress (PSS) did not differ between maternal ACE groups at baseline (*t* = − 0.58; df = 142.18; *p* = 0.562) or ultrasound 1 (*t* = − 1.46; df = 141.05; *p* = 0.148) or 2 (*t* = − 0.67; df = 140.55; *p* = 0.504).

### Testing for covariates

The only two demographic variables that met criteria to be included as covariates were race (maternal ACE group: *p* = 0.032; _wa_FAV [cm^3^/kg] at ultrasound 1: *t* = 2.28, df = 134, *p* = 0.024; _wa_FAV [cm^3^/kg] at ultrasound 2: *t* = 0.14, df = 133, *p* = 0.89) and gestational age at the first ultrasound (maternal ACE group: df = 2.08, df = 138.97, *p* = 0.039; _wa_FAV at ultrasound 1: *t* = − 2.95; df = 133; *p* = 0.004) but not at the second ultrasound (maternal ACE group: *t* = 2.39, df = 138.99, *p* = 0.018; _wa_FAV at ultrasound 2: *t* = − 1.88; df = 131; *p* = 0.062). For all analyses involving _wa_FAV as an outcome, race and time-varying gestational age were included as covariates.

### Three-way interaction of maternal ACE group, offspring sex, and time on fetal adrenal volume

**Fig. 1:**
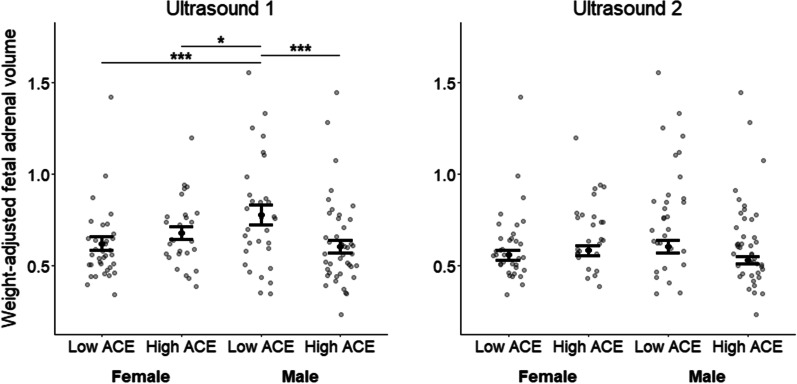
Fetal adrenal volume by maternal adverse childhood experiences (ACE) group and offspring sex. Although the three-way interaction of maternal ACE group (low vs. high), offspring sex (male vs. female), and time (ultrasound 1 vs. 2) on body weight-adjusted fetal adrenal volume (cm^3^/kg) was not significant (${\chi }^{2}$= 2.48; df = 1; *p* = .115), our primary interest was in the pairwise comparisons of the subgroups (characterized by maternal ACE and offspring sex) at ultrasounds 1 and 2. For ultrasound 1, significant differences emerged in weight − adjusted fetal adrenal volumes between low ACE boys and high ACE boys (*b* = − 0.17, *z* = − 3.75, *p* < .001), low ACE boys and low ACE girls (*b* = 0.20, *z* = 4.10, *p* < .001), and between low ACE boys and high ACE girls (*b* = − 0.11, *z* = − 2.16; *p* = .031). For the second ultrasound, no significant differences emerged for any pairwise comparisons although two findings were marginal: compared to low ACE males, high ACE males (*b* = − 0.08; 95% CI [− 0.17, 0.02]; *z* = − 1.58; *p* = .114) and low ACE females (*b* = − 0.09; 95% CI [− 0.19, 0.00]; *z* = − 1.92; *p* = .055) had marginally smaller weight-adjusted FAV. The overall model adjusts for race and gestational age at ultrasound. Bar plots display the mean and standard error of weight-adjusted fetal adrenal volume stratified by maternal ACE group and offspring sex. Maternal ACE group: low = 0–1 ACEs; high = 2 + ACEs. ***p < .001; **p < .01; *p < .05

We first tested for a three-way interaction of maternal ACE group, offspring sex, and time on _wa_FAV (cm^3^/kg), controlling for race and time-varying gestational age. Although the three-way interaction was not significant (${\chi }^{2}$= 2.48; df = 1; *p* = 0.115), our primary interest was the pairwise comparisons of the maternal ACE/offspring sex subgroups at ultrasounds 1 and 2 (Fig. 1 and Table 3), particularly given previous data suggesting that males would be more vulnerable to maternal early life stress [20, 41] and given that we did not necessarily predict changes over time in these effects. At ultrasound 1, high ACE males had a smaller _wa_FAV (*M* = 0.625; SD = 0.188) than low ACE males (*M* = 0.776; SD = 0.299; *b* = − 0.17; 95% CI [− 0.26, − 0.08]; *z* = − 3.75; *p* < 0.001), but females did not differ significantly by maternal ACE group (*b* = 0.09; 95% CI [− 0.01, 0.19]; *z* = 1.72; *p* = 0.086)—although the effect was marginal with high ACE females exhibiting larger _wa_FAV (*M* = 0.679; SD = 0.188) than low ACE females (*M* = 0.602; SD = 0.199), the opposite pattern from what was observed in males. Low (*b* = − 0.20; 95% CI [− 0.10, − 0.29]; *z* = − 4.10; *p* < 0.001) and high ACE females (*b* = − 0.11; 95% CI [− 0.21, − 0.01]; *z* = − 2.16; *p* = 0.031) had smaller _wa_FAV than low ACE males. High ACE males did not differ from low (*b* = 0.03; 95% CI [− 0.06, 0.11]; *z* = 0.57; *p* = 0.570) or high ACE females (*b* = − 0.06; 95% CI [− 0.16, 0.03]; *z* = − 1.29; *p* = 0.196) in _wa_FAV. At ultrasound 2, two marginal findings emerged: compared to low ACE males (M = 0.605; SD = 0.190), high ACE males (*M* = 0.560; SD = 0.174; *b* = − 0.08; 95% CI [− 0.17, 0.02]; *z* = − 1.58; *p* = 0.114) and low ACE females (*M* = 0.530; SD = 0.130; *b* = − 0.09; 95% CI [− 0.19, 0.00]; *z* = − 1.92; *p* = 0.055) had marginally smaller _wa_FAV. Sex differences in ACE effects do not appear to be due to confounding as males and females with the low and high ACE groups did not differ on any of the maternal and offspring variables tested in supplemental Tables 4S (low ACE group) and 5S (high ACE group).

**Table 3:** Pairwise comparisons at the first and second ultrasounds

Ultrasound	Group 1	Group 2	Estimate for group 1—group 2 (95% CI)	Z value	p value
Ultrasound 1	High ACE females	Low ACE females	0.09 (− 0.01, 0.19)	1.72	.086
**Ultrasound 1**	**Low ACE males**	**Low ACE females**	**0.20 (0.10, 0.29)**	**4.10**	** < .001**
Ultrasound 1	High ACE males	Low ACE females	0.03 (− 0.06, 0.11)	0.57	.570
**Ultrasound 1**	**High ACE females**	**Low ACE males**	** − 0.11 (− 0.21, − 0.01)**	** − 2.16**	**.031**
**Ultrasound 1**	**High ACE males**	**Low ACE males**	** − 0.17 (− 0.26, − 0.08)**	** − 3.75**	** < .001**
Ultrasound 1	High ACE males	High ACE females	− 0.06 (− 0.16, 0.03)	− 1.29	.196
Ultrasound 2	High ACE females	Low ACE females	0.06 (− 0.04, 0.15)	1.12	.262
Ultrasound 2	Low ACE males	Low ACE females	0.09 (0.00, 0.19)	1.92	.055
Ultrasound 2	High ACE males	Low ACE females	0.02 (− 0.07, 0.11)	0.44	.662
Ultrasound 2	High ACE females	Low ACE males	− 0.04 (− 0.14, 0.07)	− 0.73	.467
Ultrasound 2	High ACE males	Low ACE males	− 0.08 (− 0.17, 0.02)	− 1.58	.114
Ultrasound 2	High ACE males	High ACE females	− 0.04 (− 0.13, 0.06)	− 0.78	.437

Maternal adverse childhood experiences (ACE) group and offspring sex pairwise comparisons on fetal body weight-adjusted fetal adrenal volume (_wa_FAV). At the first ultrasound, males of mothers from the low ACE group had a larger _wa_FAV than the other three subgroups. No significant pairwise comparisons emerged at the second ultrasound although males of mothers from the low ACE group had marginally larger _wa_FAV than males of mothers from the high ACE group and females of mothers from the low ACE group. Tests with significant *p*-values are bolded

## Discussion

In a study examining the association between maternal childhood adversity and FAV (as a proxy for fetal HPA axis development), we found that, in mid-gestation, maternal ACEs were associated with significant differences in _wa_FAV in males. On average, males of mothers with a high ACE history had smaller _wa_FAV than males of mothers with a low ACE history. Not surprisingly, males of mothers with a low ACE history had larger _wa_FAV than both groups of females, in line with previous findings that male fetuses have larger fetal adrenal glands than female fetuses [30, 57]. In contrast, males of mothers with a high ACE history had _wa_FAV that was indistinguishable from both female groups. We speculate that high maternal ACEs may have dysmasculinized fetal adrenal development in males. This is in line with preclinical studies in mice and rats showing that prenatal stress or gestational exposure to corticosteroids produce male offspring dysmasculinized across a range of outcomes, such as stress responsivity [58, 59], gene expression in the brain [60], anogenital distance [20], sexual motivation and behavior [61–64], spatial memory [65], as well as play, exploratory, and risk-taking behaviors [65, 66]. The underlying mechanism for these effects may be stress hormone disruption of testosterone production during important early developmental stages of sex differentiation [67].

Although maternal ACE group effects on _wa_FAV were not significant in females, there was a trend towards the opposite pattern from what was observed in males. At both the first and the second ultrasounds, females of high ACE mothers exhibited larger _wa_FAVs than females of low ACE mothers. However, high standard deviations prohibited these mean differences from reaching statistical significance (see Additional file 1: Tables S1, S2 for means and standard deviations).

Because no prior studies have examined the relationship between preconception stress (before pregnancy) and adrenal gland development, we rely on studies assessing prenatal stress (during pregnancy) to speculate on the underlying mechanisms driving these effects, with the caveat that prenatal stress may impact the offspring differently than preconception stress. One possible mechanism is that males may be more susceptible to maternal ACE effects. Mothers carrying male fetuses already exhibit higher cortisol [68], at least during mid-gestation [69]. Furthermore, prenatal stress decreases expression of placental 11β-HSD-2 in preclinical and clinical studies [20, 70, 71], particularly when stress is chronic rather than acute [72], and some preliminary evidence in humans suggests that this effect may differ by sex [73]. Thus, maternal adversity may affect placental barriers, which may in turn impact the extent to which male fetuses may be exposed to glucocorticoids.

A second possible mechanism that could underlie our effects is the placental enzyme O-linked N-acetylglucosamine transferase (OGT), an X-linked gene expressed at lower levels in male placentas in mice and humans whose expression is further reduced by maternal prenatal stress in mice [74]. Lower OGT has been associated with reduced testosterone in male placental tissue [75], postulating a putative mechanism by which prenatal stress could result in a dysmasculinized phenotype. Although no studies have examined the impact of early life stress on OGT or OGT’s potential effects on FAV, it is possible that maternal early life stress affects OGT expression similarly to prenatal stress and could underlie our observation that high maternal ACE scores are associated with a dysmasculinized phenotype in FAV. However, this hypothesis will remain speculative until further research is conducted. Overall, fetal characteristics, such as sex, in addition to maternal factors, such as maternal ACE history, may influence maternal placental function in ways that affect fetal glucocorticoid and androgen exposure that could impact the fetal HPA axis development.

Currently, there is little data to guide the timing of when to study preconception stress effects on human fetal development. However, our study showed that findings at the second ultrasound were less robust than at the first ultrasound—although the pattern of results was the same and trended towards significance. The signal may be stronger and/or easier to detect earlier in development. One possible explanation for our significant effects at the first but not the second ultrasound is that fetal organs may become more difficult to accurately visualize using 3-D ultrasound later in gestation as the fetal bones become more ossified. This is because ossified fetal bones create more acoustic shadowing, potentially limiting the borders of fetal organs. Another possible explanation is that maternal cortisol exposure may differ across gestation depending on offspring sex. One study found that maternal cortisol was higher in mid-gestation with male fetuses and higher in late gestation with female fetuses [69]. This crossover effect, if it occurred in our sample, could have diminished the magnitude of our effects later in gestation.

### Limitations

Although our study had unique strengths in terms of measuring a novel biomarker of the fetal stress system, in retrospect, additional measures could have been useful to collect (e.g., hair cortisol in the mother during pregnancy, hair cortisol in the offspring at birth, placenta levels of 11β-HSD-2 and OGT). Although we recently published data from this cohort showing that women’s acoustic startle response did not differ by ACE during pregnancy but did in the postpartum [76], we did not measure any maternal factors that allowed us to understand the physiological impact of ACEs on the mother during pregnancy. Thus, a next important step in this research is to study the mechanisms by which maternal ACEs become biologically embedded to influence offspring development.

Given our focus on pregnant women and their offspring, ACEs could not be measured prospectively, nor did we rely on external records to confirm the occurrence of ACEs (e.g., court records). Although retrospective reporting is more susceptible to misclassification, a previous study assessing the test–retest reliability of an 8-item version of the ACE-Q [77] found its reliability to be “good” as defined by Fleiss [78] and “moderate to substantial” as defined by Landis and Koch [79].

### Perspectives and significance

Research has long established that parental life experiences impact the offspring, with studies indicating effects across the lifespan, from the time of conception to adulthood [31, 59, 80–82]. However, all previous human studies investigating outcomes in the fetus have focused on the effects of *prenatal* stress rather than *preconception* stress. To our knowledge, this study is the only one that has examined an association between a biological marker of risk in the fetus and maternal childhood adversity rather than adversity during pregnancy. Even in animal models, only one study has tested for a relationship between maternal stress and fetal adrenal gland size, but that study only examined effects of *prenatal* stress in male fetuses. Our finding of sex-specific effects of maternal ACEs on _wa_FAV emphasize the importance of considering maternal early life stress as well as offspring sex in biomedical research. Our significant findings would have been obscured if we had excluded either as a factor in our statistical analyses.

## Conclusions

In a novel study testing for associations between maternal ACEs and _wa_FAV as a marker of fetal HPA axis development, we demonstrate that maternal exposure to ACEs impacts the development of the fetal adrenal gland in a sex-specific manner. The sex differences that we observed are consistent with the limited preclinical data investigating this [20]. In males of mothers with a high ACE history, _wa_FAV was significantly smaller than in males of mothers with a low ACE history but indistinguishable from the _wa_FAV of females regardless of their mother’s ACE history. Importantly, our sample consisted of psychiatrically and medically healthy women, allowing us to isolate the impact of maternal ACEs on _wa_FAV without the confounding impact of maternal mental and physical health problems during gestation. The observed effects were presumably not due to maternal ACE effects on prenatal stress as perceived stress did not differ between maternal ACE groups at baseline or either of the two ultrasounds. Overall, our findings suggest male vulnerability to dysmasculinization of _wa_FAV in response to maternal preconception stress.

## Supplementary Information

**Additional file 1.** Supplementary Figures and Tables.

## Data Availability

Our data are publicly available at Open Science Framework (https://osf.io/bp7as/).
