# Supplementary material for: Maternal adverse childhood experiences impact fetal adrenal volume in a sex-specific manner
Source: Biol Sex Differ. 2023 Feb 17;14:7. doi: 10.1186/s13293-023-00492-0 (PMC9936707; doi:10.1186/s13293-023-00492-0)
Supplement: Supplementary file 1 — Additional file 1. Supplementary Figures and Tables. [file 13293_2023_492_MOESM1_ESM.docx]

**Supplementary Materials**

**Measuring fetal adrenal volume and body weight**

Body weight and FAV were measured using 3-D ultrasound and obtained by a specialty-trained sonographer on the Voluson E8/730 expert machines (GE Healthcare, Waukesha, WI) equipped with a transabdominal (RAB6-D) transducer. Fetal kidneys were identified in the axial plane with the fetal abdomen filling 50% to 75% of the plane in view. The angle of the volume sweep extended from above the fetal stomach to past the inferior poles of the kidneys. Data collection took approximately 30 seconds, and then the adrenal ultrasound images were archived for offline assessment with Virtual Organ Computer-Aided Analysis (VOCAL) technology (4D View, GE Healthcare) to measure FAV.

1. Kim D, Epperson CN, Ewing G, Appleby D, Sammel MD, Wang E (2016): Methodology for using 3-dimensional sonography to measure fetal adrenal gland volumes in pregnant women with and without early life stress. *J Ultrasound Med* 35: 2029–2037.

**Supplementary Figures**

**Figure 1S.** Histogram of the distribution of weight-adjusted fetal adrenal volumes for ultrasounds 1 and 2 combined.

**
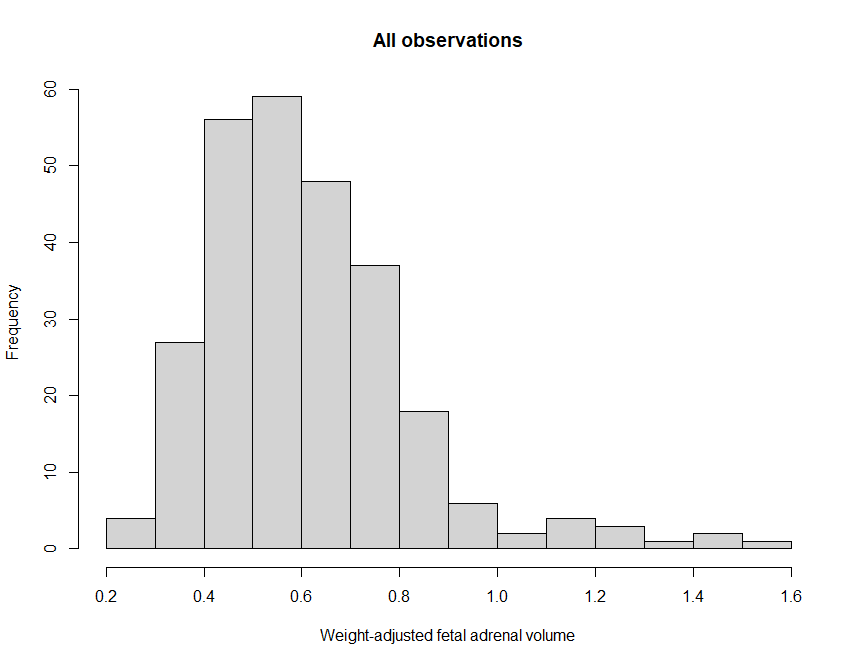
**

**Figure 2S.** Histogram of the distribution of weight-adjusted fetal adrenal volumes for ultrasounds 1 and 2 (combined) by sex.

**
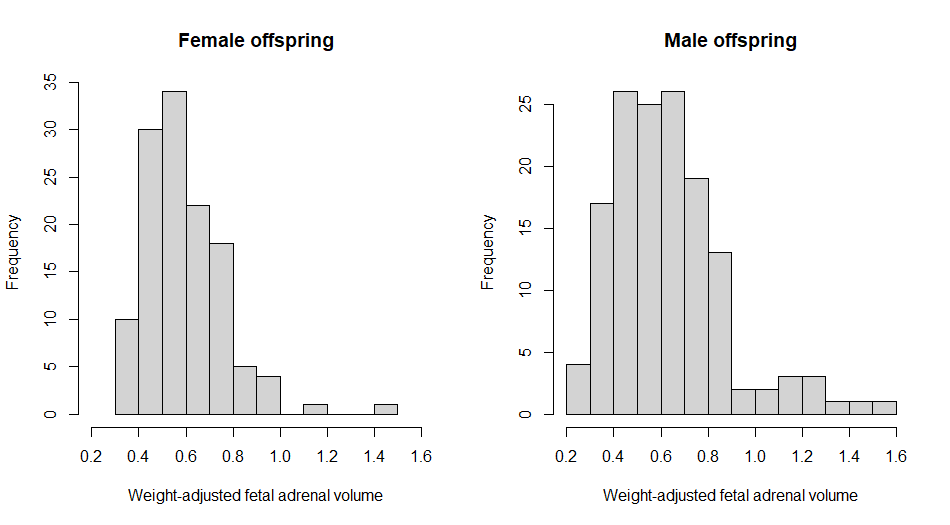
**

**Supplementary Tables**

**Table 1S. First ultrasound measures stratified by offspring sex and maternal adverse childhood experiences (ACE) group.** The significant interaction of maternal ACE group by offspring sex on fetal adrenal volume but not fetal body weight suggests that the significant interaction of maternal ACE group by offspring sex on fetal body weight-adjusted fetal adrenal volume was driven by fetal adrenal volume and not by fetal body weight.

|  | **Female** (N = 61) | | **Male** (N = 73) | | **Interaction effect: maternal ACE by offspring sex**  **p-value** |  |
| --- | --- | --- | --- | --- | --- | --- |
|  | **High ACE**  (N = 27) | **Low**  **ACE**  (N = 34) | **High**  **ACE**  (N = 42) | **Low**  **ACE**  (N = 31) |  | **Interaction effect:**  $\boldsymbol{t, df}$ |
| Gestational age (weeks) | 21.021 (1.272) | 21.592 (1.225) | 21.214 (1.32) | 21.952 (1.602) | .727 | 0.35, 1 |
| Fetal adrenal volume (cm^3^) | 0.301 (0.106) | 0.275 (0.087) | 0.288 (0.107) | 0.363 (0.11) | **.006** | 2.79, 1 |
| Fetal body weight (kg) | 0.445  (114) | 0.463  (100) | 0.473  (122) | 0.488  (117) | .940 | -0.08, 1 |
| Body weight adjusted adrenal volume (cm^3^/kg) | 0.679 (0.188) | 0.602 (0.199) | 0.625 (0.239) | 0.776  (0.299) | **.006** | 2.77, 1 |

*First ultrasound occurred at M = 21.5 (SD = 1.4) weeks gestational age. Adjusted volume was calculated by dividing adrenal volume (cm^3^) by fetal body weight (kg). Maternal ACE group: low = 0-1 ACEs; high = 2+ ACEs.*

**Table 2S. Second ultrasound measures stratified by offspring sex and maternal adverse childhood experiences (ACE) group.** The marginal interaction of maternal ACE by offspring sex on fetal adrenal volume but not fetal body weight suggests that the marginal interaction of maternal ACE by offspring sex on fetal body weight-adjusted fetal adrenal volume was driven by fetal adrenal volume and not by fetal body weight.

|  | **Female** (N = 63) | | **Male** (N = 69) | | **Interaction effect: maternal ACE by offspring sex**  **p-value** |  |
| --- | --- | --- | --- | --- | --- | --- |
|  | **High**  **ACE**  (N = 27) | **Low**  **ACE**  (N = 36) | **High**  **ACE**  (N = 41) | **Low ACE**  (N = 28) |  | **Interaction effect:**  $\boldsymbol{t, df}$ |
| Gestational age (weeks) | 29.19  (1.404) | 29.754  (1.463) | 29.121  (1.501) | 29.825  (1.168) | .780 | 0.28, 1 |
| Weeks since ultrasound 1 | 29.19  (1.404) | 29.754  (1.463) | 7.782 (1.409) | 8.033 (1.302) | .880 | -0.15, 1 |
| Fetal adrenal volume (cm^3^) | 0.841  (0.218) | 0.816  (0.216) | 0.819  (0.291) | 0.933  (0.268) | .122 | 1.56, 1 |
| Fetal body weight (kg) | 1.461  (281) | 1.555  (270) | 1.473  (271) | 1.567  (221) | .992 | -0.01, 1 |
| Body weight adjusted adrenal volume (cm^3^/kg) | 0.584  (0.143) | 0.530  (0.130) | 0.560  (0.174) | 0.605  (0.190) | .085 | 1.74, 1 |

*Second ultrasound occurred at M = 29.5 (SD = 1.4) weeks gestational age. Adjusted volume was calculated by dividing adrenal volume (cm^3^) by fetal body weight (kg). Maternal ACE group: low = 0-1 ACEs; high = 2+ ACEs.*

**Table 3S. Best fit model for defining the ACE variable.** To determine whether continuous ACEs versus dichotomized ACEs (exploring various cutoff points) produced the best fitting model (lowest Akaike Information Criterion [AIC]), we modeled the three-way interaction of maternal ACEs, offspring sex (male vs. female), and time (ultrasound 1 vs. 2) on weight-adjusted fetal adrenal volume in a linear mixed effects model with a random intercept for each participant. We found that dichotomizing ACE by a low (0-1 ACEs) and high (2+ ACEs) group led to the best fitting model based on having the lowest AIC.

|  | **ACE * sex * time** |
| --- | --- |
| **ACE variable** | **AIC** |
| Continuous | -39.1 |
| 0 vs 1+ | -57.2 |
| **0-1 vs 2+** | **-58.6** |
| 0-2 vs 3+ | -49.5 |
| 0-3 vs 4+ | -49.5 |
| 0-4 vs 5+ | -51.4 |
| 0-5 vs 6+ | -55.5 |
| 0-6 vs 7+ | -55.8 |

**Table 4S. Low maternal ACE group overall and stratified by males and females for maternal demographics, maternal psychological variables, and offspring demographics.** No statistically significant differences emerged between males and females of low ACE mothers on any of the variables tested.

|  | **Low ACE females**  (N = 39) | **Low ACE males** (N = 32) | **P-value** | |
| --- | --- | --- | --- | --- |
| **Maternal demographics** |  |  |  | |
| Maternal age | 28.9 (4.7) | 28.5 (5.5) | 0.739 | |
| BMI |  |  | 0.590 | |
| Normal/underweight | 25 (65.8%) | 18 (56.2%) |  | |
| Overweight/obese | 13 (34.2%) | 14 (43.8%) |  | |
| Missing | N = 1 | N = 0 |  | |
| Race |  |  | 0.143 | |
| African American/Black | 12 (30.8%) | 16 (50.0%) |  | |
| Caucasian/other | 27 (69.2%) | 16 (50.0%) |  | |
| Ethnicity |  |  | 0.652 | |
| Non-Hispanic/unknown | 37 (94.9%) | 29 (90.6%) |  | |
| Hispanic | 2 (5.1%) | 3 (9.4%) |  | |
| Marital status |  |  | 0.623 | |
| Married/domestic partner | 26 (66.7%) | 19 (59.4%) |  | |
| Single/divorced | 13 (33.3%) | 13 (40.6%) |  | |
| Education |  |  | 0.172 | |
| High school education or less | 7 (17.9%) | 7 (21.9%) |  | |
| Some education after high school | 4 (10.3%) | 8 (25.0%) | |  |
| College degree or more | 28 (71.8%) | 17 (53.1%) | |  |
| Income |  |  | | 0.147 |
| $25K or less | 7 (18.9%) | 13 (40.6%) | |  |
| $25K to $75K | 6 (16.2%) | 5 (15.6%) | |  |
| $75K or more | 24 (64.9%) | 14 (43.8%) | |  |
| Parity | 0.5 (0.6) | 0.7 (0.7) | | 0.331 |
| **Maternal psychological measures** |  |  | |  |
| Baseline PSS score | 13.3 (4.3) | 14.2 (7.1) | | 0.553 |
| Ultrasound 1 PSS score | 10.3 (6.7) | 9.6 (5.2) | | 0.612 |
| Ultrasound 2 PSS score | 10.0 (7.2) | 9.2 (6.4) | | 0.637 |
| Baseline EPDS score | 3.7 (3.0) | 3.7 (3.6) | | 0.998 |
| Baseline STAI state score | 26.1 (6.4) | 29.2 (9.7) | | 0.123 |
| Baseline STAI trait score | 30.0 (6.4) | 29.8 (6.5) | | 0.907 |
| **Offspring demographics** |  |  | |  |
| Gestational age (weeks) |  |  | |  |
| Baseline | 1.7 (0.3) | 1.7 (0.3) | | 0.989 |
| Ultrasound 1 | 21.6 (1.2) | 22.0 (1.6) | | 0.330 |
| Ultrasound 2 | 29.8 (1.4) | 29.8 (1.2) | | 0.983 |
| Difference from U1 to U2 | 8.3 (1.5) | 8.0 (1.4) | | 0.399 |
| Birthweight (g) | 3226 (444) | 3213 (673) | | 0.932 |
| **ACE score** |  |  | |  |
| Total ACE | 0.3 (0.5) | 0.4 (0.5) | | 0.534 |
| Any abuse | 1 (2.6%) | 3 (9.4%) | | 0.321 |
| Any neglect | 1 (2.6%) | 0 (0.0%) | | 1 |
| Any household dysfunction | 11 (28.2%) | 10 (31.2%) | | 0.800 |

**Table 5S. High maternal ACE group overall and stratified by males and females for maternal demographics, maternal psychological variables, and offspring demographics.** No statistically significant differences emerged between males and females of high ACE mothers on any of the variables tested.

|  | **High ACE females** (N = 30) | **High ACE males** (N = 46) | **P-value** |
| --- | --- | --- | --- |
| **Maternal demographics** |  |  |  |
| Maternal age | 27.5 (5.8) | 28.5 (5.3) | 0.467 |
| BMI |  |  | 0.302 |
| Normal/underweight | 10 (33.3%) | 19 (44.2%) |  |
| Overweight/obese | 20 (66.7%) | 24 (55.8%) |  |
| Missing | N = 0 | N = 3 |  |
| Race |  |  | 0.242 |
| African American/Black | 20 (66.7%) | 24 (52.2%) |  |
| Caucasian/other | 10 (33.3%) | 22 (47.8%) |  |
| Ethnicity |  |  | 1 |
| Non-Hispanic/unknown | 28 (93.3%) | 42 (91.3%) |  |
| Hispanic | 2 (6.7%) | 4 (8.7%) |  |
| Marital status |  |  | 0.813 |
| Married/domestic partner | 13 (43.3%) | 18 (39.1%) |  |
| Single/divorced | 17 (56.7%) | 28 (60.9%) |  |
| Education |  |  | 0.833 |
| High school education or less | 8 (26.7%) | 15 (32.6%) |  |
| Some education after high school | 12 (40.0%) | 16 (34.8%) |  |
| College degree or more | 10 (33.3%) | 15 (32.6%) |  |
| Income |  |  | 0.750 |
| $25K or less | 11 (36.7%) | 21 (45.7%) |  |
| $25K to $75K | 12 (40.0%) | 15 (32.6%) |  |
| $75K or more | 7 (23.3%) | 10 (21.7%) |  |
| Parity | 0.8 (1.1) | 0.8 (1.0) | 0.928 |
| **Maternal psychological measures** |  |  |  |
| Baseline PSS score | 13.0 (7.2) | 15.1 (6.5) | 0.202 |
| Ultrasound 1 PSS score | 10.7 (6.8) | 12.2 (7.3) | 0.379 |
| Ultrasound 2 PSS score | 9.4 (6.1) | 11.0 (7.3) | 0.300 |
| Baseline EPDS score | 5.2 (5.2) | 5.2 (4.2) | 0.967 |
| Baseline STAI state score | 29.8 (11.4) | 30.4 (9.6) | 0.816 |
| Baseline STAI trait score | 32.3 (9.4) | 33.8 (9.9) | 0.501 |
| **Offspring demographics** |  |  |  |
| Gestational age (weeks) |  |  |  |
| Baseline | 1.6 (0.3) | 1.7 (0.4) | 0.086 |
| Ultrasound 1 | 21.1 (1.4) | 21.4 (1.5) | 0.351 |
| Ultrasound 2 | 29.2 (1.4) | 29.2 (1.5) | 0.980 |
| Difference from U1 to U2 | 8.2 (1.6) | 7.8 (1.4) | 0.378 |
| Birthweight (g) | 3312 (451) | 3322 (494) | 0.935 |
| ACE score |  |  |  |
| Total ACE | 3.6 (1.7) | 3.6 (1.7) | 0.994 |
| Any abuse | 21 (70.0%) | 29 (63.0%) | 0.624 |
| Any neglect | 15 (50.0%) | 17 (37.0%) | 0.343 |
| Any household dysfunction | 26 (86.7%) | 38 (82.6%) | 0.754 |
